# Coexpression of VEGF-C and COX-2 and its association with lymphangiogenesis in human breast cancer

**DOI:** 10.1186/1471-2407-8-4

**Published:** 2008-01-13

**Authors:** Xiao-Hua Zhang, Du-Ping Huang, Gui-Long Guo, Guo-Rong Chen, Hu-Xiang Zhang, Li Wan, Sheng-Ying Chen

**Affiliations:** 1 Department of Oncology, The First Affiliated Hospital of Wenzhou Medical College, Wenzhou, 325000, China; 2 Department of Pathology, The First Affiliated Hospital of Wenzhou Medical College, Wenzhou, 325000, China; 3 Department of stomatology, The First Affiliated Hospital of Wenzhou Medical College, Wenzhou, 325000, China

## Abstract

**Background:**

Lymphangiogenesis has become a new research frontier in tumor metastasis since the discovery of reliable lymphatic markers that have allowed observation and isolation of lymphatic endothelium. Cyclooxygenase-2 (COX-2) has been reported to be involved in the critical steps in carcinogenesis. However, possible role of COX-2 in lymphangiogenesis and lymphatic metastasis is still poorly understood. In present study, we aimed to investigate the relationship between vascular endothelial growth factor-C (VEGF-C) and COX-2 in human breast cancer, and correlations with lymphangiogenesis and prognosis.

**Methods:**

Tissue samples of primary tumors from 70 patients undergoing intentionally curative surgical resections for breast cancer were immunohistochemically examined for VEGF-C, COX-2, and D2-40 expressions. The association between COX-2 and VEGF-C expressions and clinicopathological parameters as well as prognosis were analysised. To demonstrate the presence of proliferating lymphatic endothelial cells, 10 random cases with high LVD counts were selected for D2-40/Ki-67 double immunostaining.

**Results:**

A significant correlation was found between the expression of VEGF-C and COX-2 (*r *= 0.529, *P *< 0.001), and both elevated VEGF-C expression and elevated COX-2 expression were associated with higher lymph vessel density (LVD), lymph node metastasis and D2-40 positive lymphatic invasion (LVI) as well as worse disease free survival (DFS) and overall survival (OS) in a univariate analysis. In the double immunostain for the lymph vessel marker D2-40 and the proliferation marker Ki-67, the results confirmed Ki-67-positive nuclei in a proportion of lymph vessel endothelial cells.

**Conclusion:**

There is indeed lymphangiogenesis in breast cancer, the most compelling evidence being the presence of proliferating lymphatic endothelial cells. VEGF-C and COX-2 are coexpressed and significantly associated with lymphangiogenesis and prognosis in invasive breast cancer. Suggesting COX-2 may up-regulate VEGF-C expression and thus promote lymph node metastasis via lymphangiogenesis pathway in human breast cancer.

## Background

The lymphatic vasculature is an important route for the metastatic spread of human cancer. And the presence of tumour foci in lymph nodes is the most important adverse prognostic factor in a variety of human cancers. Recently, lymphangiogenesis, the formation of new lymphatic vessels, has become a new research frontier in tumor metastasis since the discovery of the two major lymphatic vessel growth factors-C (VEGF-C) and -D (VEGF-D), as well as reliable lymphatic markers that have allowed observation and isolation of lymphatic endothelium[1]. VEGF-C, a member of the VEGF family, has been shown to promote tumour lymphangiogenesis, the spreading of tumour cells to lymph nodes in various animal models of cancer[2,3]. Furthermore, the elevation of VEGF-C appears to correlate with lymph node metastasis in numerous human cancers including breast cancer [4].

Cyclooxygenase-2 (COX-2), the inducible isoform of prostaglandin H synthase, has been reported to be significantly overexpressed in a variety of human malignancies including breast cancer, and was identified to be involved in the critical steps in carcinogenesis [5-7]. However, possible role of COX-2 in lymphangiogenesis and lymphatic metastasis is still poorly understood. Recently, it was reported that COX-2 up-regulated VEGF-C and promotes lymphangiogenesis in human lung adenocarcinoma[8] and oesophageal adenocarcinoma [9,10] as well as in the head and neck [11]. However, data is still scarce in breast cancer and it is necessary to provide more documents to increase the dataset.

In present study, we aimed to investigate the role of COX-2 immunohistochemical expression in lymphangiogenesis, VEGF-C expression and D2-40 positive lymphatic vessel invasion (LVI) as well as prognosis in a series of archival human invasive breast cancer samples.

## Methods

### Patients and specimens

Paraffin-embedded archival specimens from 70 patients with unilateral, invasive breast cancer, who were diagnosed and treated in the Department of Oncology, The First Affiliated Hospital of Wenzhou Medical College, from Januanry 2000 to October 2001, were included in the study. We excluded patients with *in situ *carcinoma, distant metastases at the time of the diagnosis, synchronous or metachronous bilateral breast cancer, malignancy other than breast cancer in history, and the patients who had received neoadjuvant chemotherapy or radiation therapy before surgery, which left 70 patients for the analysis. All patients had received mastectomy with dissection of axillary lymph nodes, containing at least 10 nodes.

Routine histological examination was performed with hematoxylin-eosin staining. All carcinomas were classified in accordance with the criteria of the World Health Organization and were recorded as invasive ductal or invasive lobular as well as invasive medullary-like carcinomas. The combined histological grade was obtained according to a modified Scarff-Bloom-Richardson histological grading system with guidelines as suggested by Nottingham City Hospital pathologists [12]. Tumor size and lymph node status were evaluated separately. The clinicopathological characteristics of the series are shown in Table 1.

**Table 1:** Correlation of clinicopathologic parameters with VEGF-C and COX-2 expressions.

		VEGF-C Expression			COX-2 Expression		
					
Factor	No. of cases	Low	High	P value	Low	High	P value
LVD		9.63 ± 5.09	12.83 ± 5.27	0.007^a^	9.17 ± 5.07	11.96 ± 5.32	0.012^a^
Age							
≤ 50	40	23	17	0.944	12	28	0.383
> 50	30	17	13		12	18	
Tumor size							
≤ 2	28	18	10	0.324	10	18	0.837
> 2	42	22	20		14	28	
Hisyological grade							
G1 and G2	41	31	10	< 0.001	17	24	0.132
G3	29	9	20		7	22	
Lymph node metastasis							
Positive	32	13	19	0.010	6	26	0.012
Negative	38	27	11		18	20	
Histology							
IDS, NOS	59	32	27	0.420	19	40	0.614
Others	11	8	3		5	6	
ER							
Positive	54	32	22	0.511	22	32	0.037
Negative	16	8	8		2	14	
PR							
Positive	46	27	19	0.716	19	27	0.087
Negative	24	13	11		5	19	
c-erbB-2							
Positive	13	6	7	0.375	2	11	0.205
Negative	57	34	23		22	35	
LVI							
Positive	25	10	15	0.031	4	21	0.016
Negative	45	30	15		20	25	

a Analysis by Mann-Whitney U test.

### Immunohistochemistry

Sections (4 um) of paraffin-embedded tissue block were rehydrated by sequential immersion in xylene, graded ethanol and water, and then they were incubated in 3% hydrogen peroxide methanol for 5 min, and followed by using a microwave oven for antigen retrieval. After washing in phosphate-buffered saline (PBS), the slides were exposure to 10% normal goat serum for 10 min to reduce non-specific binding, this was followed by an overnight incubation at 4°C in a humidified chamber with polyclonal rabbit antihuman VEGF-C antibody (Zymed, USA) at 1:100 dilution, and monoclonal mouse antihuman COX-2 antibody (Zymed, USA) at 1:100 dilution. The antigen-antibody reaction was visualized by Picture Plus Kit (Zymed, USA) and diaminobenzidine as the chromogen. Finally, hematoxylin was used as a counterstain. Negative controls were processed as above except for the primary antibodies were used. Sections of colon cancer known to express COX-2 and VEGF-C were used as positive controls.

A monoclonal mouse antihuman D2-40 antibody (Zymed, USA) was used for the staining of lymphatic vessels. The procedure of immunohistochemical staining of D2-40 is similar to that of the COX-2 and VEGF-C staining at a dilution of 1:100. And section from previously studied case of tonsilla known to express D2-40 was used as the positive control.

To demonstrate the presence of proliferating lymphatic endothelial cells, 10 random cases with high LVD counts (above 50 percentile) were selected for D2-40/Ki-67 double immunostaining. First, a monoclonal antibody directed at Ki-67 (DakoCytomation; dilution 1:150) was applied to the rehydrated paraffin sections for 30 minutes after antigen retrieval in citrate buffer (pH 6.0) at 98°C. Sections were incubated with EnVision+ Dual Link solution before development with diaminobenzidine (DakoCytomation). Sections were then stained with the D2-40 antibody (Zymed; dilution 1:20) for 60 minutes. EnVision System alkaline phosphatase and Fast Red chromogen (DakoCytomation) were used to visualize binding of this second antibody.

### Evaluation of staining

The evaluation of staining were performed by two investigators (Zhang and Li), who were unaware of the clinical data or the disease outcome, examined all slides independently. When the interpretation differed between the two observers, slides were revaluated for a final decision at a conference microscope.

For VEGF-C and COX-2 assessment, determination of the intensity of the immunohistochemical staining was performed according to Su *et al.*[8]. The immunostained sections were scanned by light microscopy, and all of the tumor cells were evaluated: -, negative; +, focal expression < 5% of cancer tissues; + +, focal expression in 5–20% of cancer tissues; and + + +, diffuse expression > 20% of cancer tissues. The tissue with + + and + + + staining of COX-2 or VEGF-C was classified as 'high expression group' and those with - and + staining was assigned as 'low expression group'.

Determination of lymphatic vessel density (LVD) was performed as suggested by Weidner *et al.*[13]. The immunostained sections were scanned by light-microscopy at low magnification (40×) and the areas of tissue with the greatest number of distinctly highlighted microvessels ('hot spots') were selected. LVD was then determined by counting all immunostained vessels at a total magnification of (200×) from five areas for each case.

Determination of the staining reaction was strictly confined to the 'hot spots' and the mean number of lymph vessels in each case was evaluated. LVI was considered evident if at least one tumor cell cluster was clearly visible inside the D2-40 stained vascular space [14].

### Statistics

Spearman's coefficient of correlation, Chi-squared test, and Mann-Whitney test were used as appropriate. Overall survival (OS) curves and Disease free survival (DFS) curves were obtained using the Kaplan-Meier method and compared using the log-rank test. A multivariate model using the Cox stepwise regression analysis was used to evaluate the statistical strength of independent association between covariates and DFS and/or OS. For all tests, a *P*-value less than 0.05 was considered to be significant. All *P*-values given are results of two-sided tests.

## Results

### Clinical data

The median age at diagnosis for the 70 patients was 49 years (range, 30–77 years). 57.1% (n = 40) of the patients were younger than 50 years, and 45.7% (n = 32) of the patients had lymph node metastasis at the time of surgery (Table 1). Median follow-up time for the 70 subjects was 68 months (range, 28–83 months). During this observation time, 20 patients developed recurrent disease, and 15 died from their cancer.

### VEGF-C, COX-2 and D2-40 expression in human breast cancer tissues

**Figure 1:**
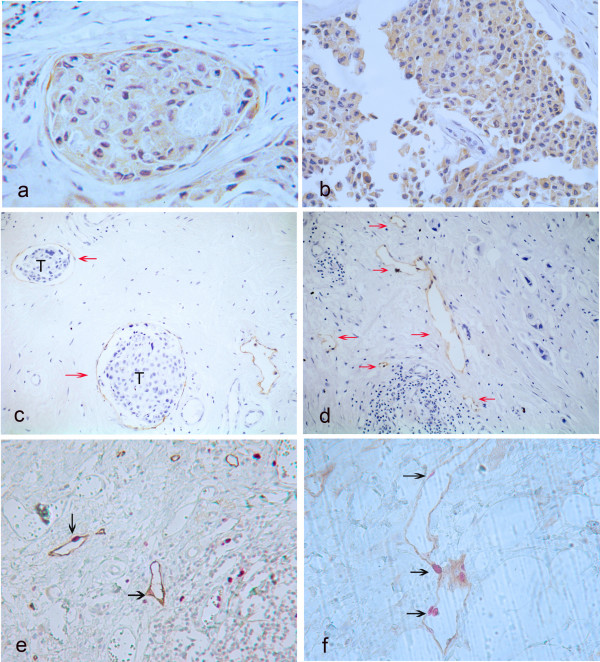
(a) : Breast cancer specimen with high vascular endothelial growth factor-C expression. Note the typical cytoplasmatic staining reaction, original magnification 400×. (b) : Breast cancer with high cyclooxygenase-2 expression. Typically granular staining was diffuse in cytoplasm of the cancer cells, original magnification 400×. (c) : D2-40-stained lymphatic vessel (arrows) with tumor cells (T) inside (lymph vessel invasion) was noted, original magnification 200×. (d) : Breast cancer specimen with a high peritumoral lymphatic vessel density (LVD), some of the lymphatic vessels stained for D2-40 are marked with arrows, original magnification 200×. (e-f): Double staining for D2-40 (brown) and Ki-67 (red) of lymph vessels; Positive staining for Ki-67 is seen in nuclei of lymphatic endothelial cells (black arrows), original magnification 400×.

Positive staining of both VEGF-C and COX-2 proteins was seen in the cytoplasm of tumor cells (Fig 1a–b). However, occasionally, normal epithelial cells and stromal components showed faint staining, particularly adjacent stromal endothelial cells for VEGF-C. High VEGF-C expression was observed in 30 of 70 tumor samples (42.8%), while high COX-2 expression occurred in 46 of the 70 tumor samples (65.7%). D2-40 expression was essentially restricted to thinwalled vessel-like structures. D2-40 positive lymphatic vessels were almost exclusively found within the tumor stroma, at the tumor's invasion front (Fig 1c). Occasional invasion of the carcinoma cells into the lymph vessels was observed (Fig 1d). Median LVD was11 microvessels/field (range, 3–26 vessels).

In the double immunostain for the lymph vessel marker D2-40 and the proliferation marker Ki-67, the evaluation of Ki-67 was performed according to Beasley *et al.*[15]. Positive staining of Ki-67 was seen in nuclei of lymphatic endothelial cells (Fig 1e–f, black arrows). Proliferating lymphatic endothelial cells were observed in 8 of the 10 cases analyzed. And, as expected, Ki-67-positive nuclei were observed in the tumor cells themselves.

### COX-2 expression correlated with VEGF-C level, lymph node metastasis and lymphatic vessel density

As showed in Table 2, there was a significant correlation between COX-2 and VEGF-C protein expression of tumor cells (*P *< 0.001, *r *= 0.529, Spearman's coefficient of correlation).

**Table 2:** Correlation between levels of cyclooxygenase-2 (COX-2) and vascular endothelial growth factor-C (VEGF-C) expression in human breast cancer.

	COX-2^a^			
VEGF-C^a^	-	+	2+	3+^b^
**-**^b^	4	6	4	1
**+**	3	8	10	4
2+	1	2	12	7
3+	0	0	2	6

a Detection of the expressing level of COX-2 and VEGF-C by immunohistochemistry.

b Intensity of immunohistochemical staining.

The associations between VEGF-C and COX-2 expressions and the clinicopathologic parameters are shown in Table 1. Both high expression of the VEGF-C and COX-2 were correlated with the presence of lymph node metastasis (*P *= 0.010, *P *= 0.012 respectively, both Chi-Square test) and LVI (*P *= 0.031, *P *= 0.016 respectively, both Chi-Square test). LVD was significantly higher in patients with high expression of VEGF-C compared to patients with low expression (*P *= 0.007, Mann-Whitney test), and there was also a similar significantly association between LVD and COX-2 expression (*P *= 0.012, Mann-Whitney test). Additionly, high expression of VEGF-C, but not of COX-2 was correlated with advanced histological grading (*P *< 0.001, Chi-Square test), and COX-2 expression was correlated with ER status (*P *= 0.037, Chi-Square test). As showed in Table 1, there was no significant association between COX-2 expression and age (*P *= 0.383, Chi-Square test), histological type (*P *= 0.614, Chi-Square test), size of primary tumor (*P *= 0.837, Chi-Square test), c-erbB-2 status (*P *= 0.205, Chi-Square test) and PR status (*P *= 0.087, Chi-Square test). Furthermore, a significant association between LVI and LVD was found (*P *= 0.002, Mann-Whitney test).

### Survival analysis

**Figure 2:**
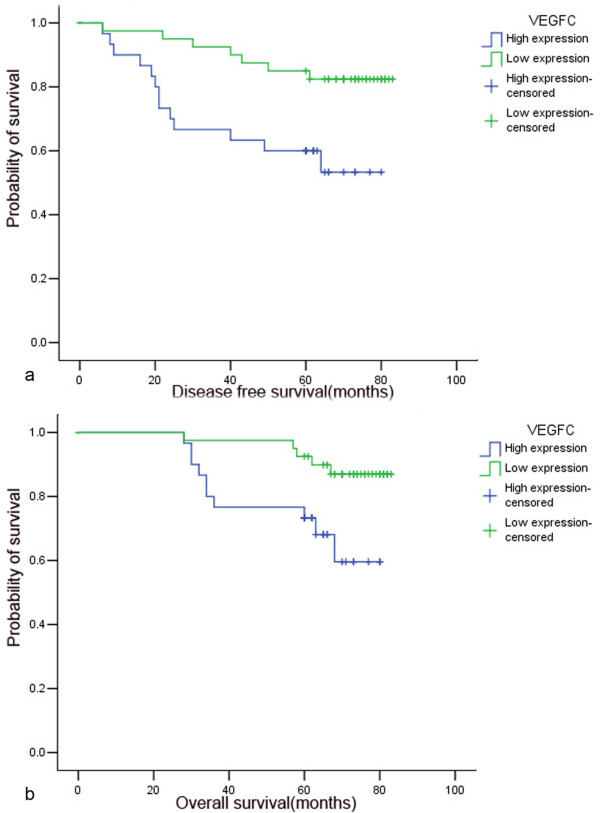
Association of VEGF-C expression with patients'prognosis in breast cancer (Kaplan-Meier method and log-rank test). High VEGF-C expression was significantly related to recurrence (a, *P *= 0.008) and death (b, *P *= 0.014).

Kaplan-Meier curves for survival are shown in Figure 2. Patients with high expression of VEGF-C showed a significantly shorter DFS (*P *= 0.008, log-rank test, Fig 2a) and OS (*P *= 0.014, log-rank test, Fig 2b) than patients with low expression. Meanwhile, patients with high expression of COX-2 was also found to have a significantly shorter DFS (*P *= 0.009, log-rank test) and OS (*P *= 0.043, log-rank test) than patients with low expression.

In Cox regression for DFS including patients'age, histological grading, VEGF-C expression, histological type, tumor size, LVD, lymph node metastasis, hormonal status, c-erbB-2, LVI and COX-2 expression, only ER (*P *= 0.045), LVI (*P *= 0.025), c-erbB-2 status (*P *= 0.007) and LVD (*P *= 0.015) remained as independent prognostic factors. However, based on multivariate Cox regression analysis for OS, only c-erbB-2 status (*P *= 0.028) and LVD (*P *= 0.002) were identified as the independent prognostic factors (Table 3).

**Table 3:** The multivariate Cox regression analysis for DFS and OS in 70 patients with invasive breast cancer.

	Significance Multivariate P (Cox regression)	95%Confidence Interval	Hazard ratio
Disease-free survival			
LVD	0.015*	1.289–11.007	3.766
ER	0.045*	0.134–0.976	0.361
c-erbB-2	0.007*	1.509–13.731	4.552
LVI	0.025*	1.162–9.648	3.348
VEGF-C expression	0.167		
COX-2 expression	0.330		
Overall survival			
LVD	0.002*	2.021–20.595	6.452
c-erbB-2	0.028*	1.142–10.199	3.413
VEGF-C expression	0.155		
COX-2 expression	0.375		

*Statistically significant (Cox regression).

## Discussion

COX-2 is a pleiotropic enzyme that mediates many physiological functions in breast cancer progression such as inhibition of cell apoptosis, increased cell motility, as well as angiogenesis [5,7]. Costa *et al.*[16] reported that COX-2 overexpression was significantly associated with lymph node metastasis in human breast cancer. However, whether COX-2 contributes to the formation of new lymphatic vessels is still little known. In the present study, elevated COX-2 expression was positively correlated with LVD and LVI. And the expression of COX-2 protein was significantly higher in lymph node-positive group than in the node-negative group. Which may suggest COX-2 promote the formation of lymphatic vessels and lymph node metastasis. In addition, univariate analysis demonstrated that high COX-2 expression was negatively associated with both DFS and OS. The result provides an explanation that patients with high COX-2 expression were more likely to have poor prognosis than low expression patients, possibly resulting from COX-2-derived lymphangiogenesis in human breast cancer.

Research on tumor lymphangiogenesis has lagged behind that of angiogenesis because of the lack of a specific lymphatic marker and the absence of detailed knowledge concerning the molecular mechanisms of lymphangiogenesis [1]. Recently, D2-40 antibody was reported to detect a fixation-resistant epitope on a 40 kDa O-linked sialoglycoprotein expressed in lymphatic endothelium but not blood vessels, and can be used to assess lymphangiogenesis specifically in conventionally processed formalin-fixed and paraffin-embedded tissue specimens [17,18]. The present study clearly demonstrated that D2-40 reacted with the endothelium of lymphatic vessels, which are covered with flattened endothelial cells, and does not react with endothelial cells of blood vessels. Suggesting D2-40 is a new selective marker of lymphatic endothelial cells. In this study, D2-40 immunohistochemistry revealed that lymph vessels were restricted to the endothelium, almost exclusively in peri-tumoral lesions but not in intra-tumoral lesions. Our results are consistent with previously reported studies [19-21], and might be explained by a rising interstitial pressure caused by an increase in the size of lesion or by the lack of intratumoral lymphangiogenesis in breast cancer. Indicating that peritumoural lymphatic vessels are important for the process of metastatic spread while intratumoural lymphatic vessels are nonfunctional [21,22].

In the present study, we carried out double immunostaining with antibodies to D2-40 and Ki-67 to observe the occurrence of dividing nuclei among lymphatic endothelial cells. The results confirmed Ki-67-positive nuclei in a proportion of lymph vessel endothelial cells, suggesting that there is indeed lymphangiogenesis in breast cancer, the most compelling evidence being the presence of proliferating lymphatic endothelial cells.

Experimental murine tumour models have demonstrated a role for VEGF-C in tumour lymphangiogenesis and the subsequent formation of lymph node metastasis [23]. Here we show that increased VEGF-C expression was associated with lymph node metastasis, higher LVD and LVI in human breast cancer. Our results suggest VEGF-C is a potent enhancer of tumor lymphangiogenesis, leading to increased metastatic spread of breast cancer cells to lymph nodes. However, Kinoshita *et al.*[24] did not obtain a significant association between VEGF-C expression and lymph node metastasis. The difference between our findings and those reported by Kinoshita *et al.*[24] may be due to the use of different antibodies and the different number of the cases as well as evaluation method of immunohistochemistry.

In the present study, a significant association between increased VEGF-C expression and advanced histological grading was found, suggesting that poorly differentiated tumor cells may be more capable to secrete VEGF-C, which induced lymphangiogenesis in breast cancer.

The secretion of VEGF-C and VEGF-D by some tumours could induce the activation of their receptor, VEGFR-3 on the vascular endothelium and thereby inducing the formation of new lymphatic vessels. However, little is currently known about the factors that make dome tumours secret these lymphangiogenic factors [25]. In the present study, a significant positive correlation between COX-2 and VEGF-C protein expression of tumor cells was seen, which confirm to the previous studies [8-11,26], suggesting a lymphangiogenesis pathway that COX-2 may up-regulate VEGF-C expression and thus the formation of new lymphatic vessels in human breast cancer. However, the correlation is much weaker compared with the result reported by Timoshenko *et al.*[26] (correlation coefficients 0.553 vs 0.940). The discrepancy may be due to use of different methods and different number of tumors studied. For example, COX-2 and VEGF-C mRNA expression could also be observed in normal breast epithelium [27,28], RT-PCR assay could not to reveal the two molecules expression exactly if microdissection was not performed, as well as the possibility that stromal cells and/or immigrant leukocytes may also be the source of both molecules except for the breast cancer cells. Recently, HIF-1a was also found to have a possible role in tumor lymphangiogenesis through the regulation of VEGF-C in human esophageal cancer [29]. This suggests COX-2 is an important, but not the only VEGF-C upstream regulator in tumor lymphangiogenesis. COX-2 and VEGF-C expression should be examined in the context of other proposed lymphangiogenic molecules such as HIF-1a in further investigation.

## Conclusion

Our study showed that COX-2 and VEGF-C may play an important role in tumor metastasis. COX-2 may be up-regulated by VEGF-C expression in order to promote lymphangiogenesis in human breast cancer. It is a considerable speculation that a COX-2 inhibitor prevents lymph node metastasis of breast cancer in clinical use.

## Competing interests

The author(s) declare that they have no competing interests.

## Authors' contributions

XHZ and DPH designed and interpreted the experiments. DPH performed most of the experiments. GLG and GRC assisted with design/execution of some experiments. HXZ and LW carried out the immunoassays. DPH, SYC and XHZ wrote the manuscript. All authors read and approved the final the manuscript.

## Pre-publication history

The pre-publication history for this paper can be accessed here:


